# Correction: A navigational risk evaluation of ferry transport: Continuous risk management matrix based on fuzzy Best-Worst Method

**DOI:** 10.1371/journal.pone.0320794

**Published:** 2025-03-17

**Authors:** Linh Thi Pham, Long Van Hoang

There is an error in affiliation 2 for author Long Van Hoang. The correct affiliation 2 is: Faculty of Management, Ho Chi Minh City University of Law, Ho Chi Minh City, Vietnam
