# Supplementary material for: A navigational risk evaluation of ferry transport: Continuous risk management matrix based on fuzzy Best-Worst Method
Source: PLoS One. 2024 Sep 3;19(9):e0309667. doi: 10.1371/journal.pone.0309667 (PMC11371230; doi:10.1371/journal.pone.0309667)
Supplement: S1 File — (ZIP) [file pone.0309667.s001.zip › Raw material for severity.pdf]

Data sources for five constructs

Respondent 1

	C1	C2	C3	C4	C5
C5	7	1	5	7	1
C1	1	7	5	3	7

Respondent 2

	C1	C2	C3	C4	C5
C3	7	3	1	7	5
C1	1	1	7	1	5

Respondent 3

	C1	C2	C3	C4	C5
C5	1	7	5	9	1
C2	1	1	7	7	7

Respondent 4

	C1	C2	C3	C4	C5
C1	1	7	1	7	5
C5	5	5	5	5	1

Respondent 5

	C1	C2	C3	C4	C5
C2	9	1	5	3	9
C5	1	9	7	3	1

Respondent 6

	C1	C2	C3	C4	C5
C5	3	9	9	9	1
C1	1	3	9	5	3

Respondent 7

	C1	C2	C3	C4	C5
C3	1	7	1	1	3
C1	1	1	1	3	9

Respondent 8

	C1	C2	C3	C4	C5
C3	3	5	1	3	1
C5	9	9	1	3	1

Respondent 9

	C1	C2	C3	C4	C5
C4	5	9	3	1	9
C2	7	1	1	9	5

Respondent 10

	C1	C2	C3	C4	C5
C5	1	7	5	9	1
C2	1	1	7	7	7

Respondent 11

	C1	C2	C3	C4	C5
C3	1	3	1	7	5
C1	1	1	1	3	3

Respondent 12

	C1	C2	C3	C4	C5
C1	1	3	7	1	7
C3	7	5	1	7	5

Respondent 13

	C1	C2	C3	C4	C5
C1	1	1	9	9	3
C4	9	9	7	1	9

Respondent 14

	C1	C2	C3	C4	C5
C5	7	7	5	9	1
C3	3	3	1	1	5

Respondent 15

	C1	C2	C3	C4	C5
C3	7	7	1	3	1
C5	7	5	1	7	1

Respondent 16

	C1	C2	C3	C4	C5
C1	1	7	9	7	3
C3	9	7	1	7	9

Respondent 17

	C1	C2	C3	C4	C5
C1	1	5	5	9	1
C5	1	5	1	1	1

Respondent 18

	C1	C2	C3	C4	C5
C4	3	9	5	1	3
C5	1	3	1	3	1

Respondent 19

	C1	C2	C3	C4	C5
C5	3	5	7	9	1
C1	1	7	7	1	3

Respondent 20

	C1	C2	C3	C4	C5
C5	9	5	1	5	1
C1	1	1	7	7	9

Respondent 21

	C1	C2	C3	C4	C5
C5	9	3	7	3	1
C1	1	3	9	9	9

Respondent 22

	C1	C2	C3	C4	C5

C1	1	5	3	1	7
C3	3	1	1	3	1

Respondent 23

	C1	C2	C3	C4	C5
C5	1	7	5	9	1
C2	1	1	7	7	7

Respondent 24

	C1	C2	C3	C4	C5
C4	3	7	7	1	7
C3	7	1	1	7	7

Respondent 25

	C1	C2	C3	C4	C5
C1	1	7	9	9	7
C5	7	7	7	1	1

Respondent 26

	C1	C2	C3	C4	C5
C3	1	1	1	5	1
C5	5	9	1	7	1

Respondent 27

	C1	C2	C3	C4	C5
C1	1	1	3	3	9
C3	3	3	1	3	9

Respondent 28

	C1	C2	C3	C4	C5
C5	9	5	3	5	1
C3	3	1	1	3	3

Data sources for the HF construct

Respondent 1

	C1	C2	C3	C4
C4	7	3	5	1
C2	9	1	7	3

Respondent 2

	C1	C2	C3	C4
C2	9	1	5	7
C4	1	7	9	1

Respondent 3

	C1	C2	C3	C4
C1	1	7	5	3
C4	3	5	5	1

Respondent 4

	C1	C2	C3	C4
C3	7	5	1	7
C4	7	1	7	1

Respondent 5

	C1	C2	C3	C4
C1	1	7	1	3
C3	1	5	1	9

Respondent 6

	C1	C2	C3	C4
C2	1	1	3	7
C3	1	3	1	9

Respondent 7

	C1	C2	C3	C4
C1	1	9	5	5
C4	5	1	7	1

Respondent 8

	C1	C2	C3	C4
C4	7	3	5	1
C2	9	1	7	3

Respondent 9

	C1	C2	C3	C4
C4	5	1	7	1
C2	9	1	3	1

Respondent 10

	C1	C2	C3	C4
C4	7	3	5	1
C2	9	1	7	3

Respondent 11

	C1	C2	C3	C4
C1	1	1	5	7
C4	7	3	7	1

Respondent 12

	C1	C2	C3	C4
C4	5	5	9	1
C1	1	5	5	5

Respondent 13

	C1	C2	C3	C4
C4	1	7	3	1
C1	1	7	7	1

Respondent 14

	C1	C2	C3	C4
C4	3	7	7	1
C2	9	1	3	7

Respondent 15

	C1	C2	C3	C4
C1	1	3	5	3
C4	3	3	9	1

Respondent 16

	C1	C2	C3	C4
C4	1	5	1	1
C1	1	1	1	1

Respondent 17

	C1	C2	C3	C4
C4	9	5	5	1
C1	1	9	5	9

Respondent 18

	C1	C2	C3	C4
C4	5	1	7	1
C2	9	1	3	1

Respondent 19

	C1	C2	C3	C4
C1	1	1	3	5
C4	5	5	9	1

Respondent 20

	C1	C2	C3	C4
C4	3	5	7	1
C1	1	9	5	3

Respondent 21

	C1	C2	C3	C4
C4	7	5	3	1
C2	5	1	5	5

Respondent 22

	C1	C2	C3	C4

C4	5	1	7	1
C2	9	1	3	1

Respondent 23

	C1	C2	C3	C4
C4	9	5	1	1
C1	1	1	1	9

Respondent 24

	C1	C2	C3	C4
C3	7	9	1	9
C4	3	7	9	1

Respondent 25

	C1	C2	C3	C4
C1	1	3	1	7
C3	1	5	1	7

Respondent 26

	C1	C2	C3	C4
C3	7	9	1	9
C4	3	7	9	1

Respondent 27

	C1	C2	C3	C4
C4	9	5	3	1
C1	1	5	7	9

Respondent 28

	C1	C2	C3	C4
C4	9	7	9	1
C3	3	5	1	9

Data sources for the NE construct

Respondent 1

	C1	C2	C3	C4
C2	3	1	3	5
C1	1	3	7	5

Respondent 2

	C1	C2	C3	C4
C4	7	9	5	1
C1	1	3	5	7

Respondent 3

	C1	C2	C3	C4
C4	7	3	5	1
C2	9	1	7	3

Respondent 4

	C1	C2	C3	C4
C3	9	1	1	7
C4	3	9	7	1

Respondent 5

	C1	C2	C3	C4
C4	7	3	5	1
C2	9	1	7	3

Respondent 6

	C1	C2	C3	C4
C1	1	1	7	7
C4	7	3	9	1

Respondent 7

	C1	C2	C3	C4
C2	9	1	9	5
C3	5	9	1	7

Respondent 8

	C1	C2	C3	C4
C1	1	9	1	5
C4	5	7	5	1

Respondent 9

	C1	C2	C3	C4
C3	3	9	1	7
C4	7	9	7	1

Respondent 10

	C1	C2	C3	C4
C4	1	9	1	1
C1	1	1	7	1

Respondent 11

	C1	C2	C3	C4
C4	3	3	9	1
C2	7	1	5	3

Respondent 12

	C1	C2	C3	C4
C4	7	3	5	1
C2	9	1	7	3

Respondent 13

	C1	C2	C3	C4
C4	3	3	5	1
C3	9	1	1	5

Respondent 14

	C1	C2	C3	C4
C4	7	3	9	1
C4	5	5	1	1

Respondent 15

	C1	C2	C3	C4
C4	7	3	5	1
C2	9	1	7	3

Respondent 16

	C1	C2	C3	C4
C4	1	5	5	1
C2	9	1	5	5

Respondent 17

	C1	C2	C3	C4
C4	3	1	7	1
C2	3	1	5	1

Respondent 18

	C1	C2	C3	C4
C4	7	3	5	1
C2	9	1	7	3

Respondent 19

	C1	C2	C3	C4
C2	7	1	9	3
C4	9	3	7	1

Respondent 20

	C1	C2	C3	C4
C3	9	3	1	1
C1	1	9	9	9

Respondent 21

	C1	C2	C3	C4
C4	7	1	1	1
C1	1	5	9	7

Respondent 22

	C1	C2	C3	C4

C4	7	3	5	1
C2	9	1	7	3

Respondent 23

	C1	C2	C3	C4
C2	5	1	3	9
C1	1	5	5	9

Respondent 24

	C1	C2	C3	C4
C3	1	3	1	3
C4	7	9	3	1

Respondent 25

	C1	C2	C3	C4
C4	1	7	5	1
C2	1	1	7	7

Respondent 26

	C1	C2	C3	C4
C4	7	3	5	1
C3	7	1	1	5

Respondent 27

	C1	C2	C3	C4
C2	5	1	3	1
C4	1	1	5	1

Respondent 28

	C1	C2	C3	C4
C2	7	1	3	3
C4	9	3	1	1

Data sources for the PR construct

Respondent 1

	C1	C2	C3	C4
C3	5	7	1	5
C2	7	1	7	9

Respondent 2

	C1	C2	C3	C4
C4	1	7	5	1
C1	1	3	7	1

Respondent 3

	C1	C2	C3	C4
C3	9	9	1	3
C4	7	7	3	1

Respondent 4

	C1	C2	C3	C4
C4	3	5	7	1
C1	1	5	5	3

Respondent 5

	C1	C2	C3	C4
C1	1	7	9	7
C4	7	3	9	1

Respondent 6

	C1	C2	C3	C4
C1	1	1	9	3
C4	3	9	5	1

Respondent 7

	C1	C2	C3	C4
C1	1	1	1	9
C4	9	1	9	1

Respondent 8

	C1	C2	C3	C4
C4	7	3	9	1
C2	3	1	7	3

Respondent 9

	C1	C2	C3	C4
C4	1	5	5	1
C2	3	1	3	5

Respondent 10

	C1	C2	C3	C4
C1	1	5	1	9
C4	9	7	1	1

Respondent 11

	C1	C2	C3	C4
C4	9	1	5	1
C2	7	1	1	1

Respondent 12

	C1	C2	C3	C4
C3	3	9	1	3
C1	1	5	3	9

Respondent 13

	C1	C2	C3	C4
C4	5	7	7	1
C2	9	1	5	7

Respondent 14

	C1	C2	C3	C4
C4	7	7	3	1
C1	1	5	5	7

Respondent 15

	C1	C2	C3	C4
C1	1	7	1	3
C4	3	9	5	1

Respondent 16

	C1	C2	C3	C4
C2	9	1	1	1
C1	1	9	5	5

Respondent 17

	C1	C2	C3	C4
C4	1	3	7	1
C1	1	9	1	1

Respondent 18

	C1	C2	C3	C4
C4	1	7	5	1
C1	1	3	7	1

Respondent 19

	C1	C2	C3	C4
C4	1	3	1	1
C2	3	1	1	3

Respondent 20

	C1	C2	C3	C4
C4	7	3	5	1
C2	3	1	5	3

Respondent 21

	C1	C2	C3	C4
C4	7	9	9	1
C3	1	1	1	9

Respondent 22

	C1	C2	C3	C4

C4	5	7	1	1
C1	1	9	9	5

Respondent 23

	C1	C2	C3	C4
C1	1	7	7	1
C4	1	5	9	1

Respondent 24

	C1	C2	C3	C4
C4	1	7	5	1
C1	1	3	7	1

Respondent 25

	C1	C2	C3	C4
C4	7	3	5	1
C2	9	1	7	3

Respondent 26

	C1	C2	C3	C4
C2	9	1	1	3
C4	3	3	5	1

Respondent 27

	C1	C2	C3	C4
C1	1	1	9	9
C4	9	5	3	1

Respondent 28

	C1	C2	C3	C4
C4	7	3	5	1
C2	9	1	7	3

Data sources for the VD construct

Respondent 1

	C1	C2	C3	C4
C3	3	5	1	9
C4	1	9	9	1

Respondent 2

	C1	C2	C3	C4
C1	1	5	7	7
C4	7	3	1	1

Respondent 3

	C1	C2	C3	C4
C4	9	9	5	1
C2	1	1	1	9

Respondent 4

	C1	C2	C3	C4
C3	5	5	1	3
C4	9	9	3	1

Respondent 5

	C1	C2	C3	C4
C4	7	1	9	1
C3	5	3	1	9

Respondent 6

	C1	C2	C3	C4
C3	9	1	1	5
C4	9	3	5	1

Respondent 7

	C1	C2	C3	C4
C4	9	9	5	1
C2	1	1	1	9

Respondent 8

	C1	C2	C3	C4
C3	3	9	1	7
C4	1	5	7	1

Respondent 9

	C1	C2	C3	C4
C4	1	9	3	1
C1	1	5	7	1

Respondent 10

	C1	C2	C3	C4
C2	7	1	5	3
C4	9	3	9	1

Respondent 11

	C1	C2	C3	C4
C4	3	3	7	1
C1	1	9	9	3

Respondent 12

	C1	C2	C3	C4
C2	5	1	7	3
C4	3	3	9	1

Respondent 13

	C1	C2	C3	C4
C4	1	3	1	1
C3	7	1	1	1

Respondent 14

	C1	C2	C3	C4
C2	1	1	1	1
C4	1	1	9	1

Respondent 15

	C1	C2	C3	C4
C4	9	9	7	1
C2	3	1	7	9

Respondent 16

	C1	C2	C3	C4
C1	1	9	1	9
C4	9	9	7	1

Respondent 17

	C1	C2	C3	C4
C2	3	1	5	1
C4	1	1	3	1

Respondent 18

	C1	C2	C3	C4
C4	9	9	5	1
C2	1	1	1	9

Respondent 19

	C1	C2	C3	C4
C1	1	1	9	5
C4	5	9	5	1

Respondent 20

	C1	C2	C3	C4
C4	3	1	7	1
C3	5	7	1	7

Respondent 21

	C1	C2	C3	C4
C4	5	7	1	1
C3	7	3	1	1

Respondent 22

	C1	C2	C3	C4

C2	5	1	9	9
C4	9	9	5	1

Respondent 23

	C1	C2	C3	C4
C4	1	5	9	1
C1	1	5	5	1

Respondent 24

	C1	C2	C3	C4
C4	1	7	5	1
C1	1	3	7	1

Respondent 25

	C1	C2	C3	C4
C1	1	1	9	5
C4	5	9	5	1

Respondent 26

	C1	C2	C3	C4
C4	1	7	5	1
C1	1	3	7	1

Respondent 27

	C1	C2	C3	C4
C2	3	1	9	3
C1	1	3	7	1

Respondent 28

	C1	C2	C3	C4
C4	1	7	5	1
C1	1	3	7	1

Data sources for the EE construct

Respondent 1

	C1	C2	C3	C4
C4	1	3	1	1
C1	1	9	7	1

Respondent 2

	C1	C2	C3	C4
C3	7	1	1	5
C4	1	3	5	1

Respondent 3

	C1	C2	C3	C4
C1	1	5	7	9
C2	5	1	5	1

Respondent 4

	C1	C2	C3	C4
C4	5	3	7	1
C2	3	1	9	3

Respondent 5

	C1	C2	C3	C4
C4	7	5	1	1
C3	5	7	1	1

Respondent 6

	C1	C2	C3	C4
C4	5	3	5	1
C2	1	1	5	3

Respondent 7

	C1	C2	C3	C4
C4	7	3	3	1
C3	3	1	1	3

Respondent 8

	C1	C2	C3	C4
C3	1	9	1	3
C4	7	7	3	1

Respondent 9

	C1	C2	C3	C4
C2	1	1	3	5
C1	1	1	7	9

Respondent 10

	C1	C2	C3	C4
C3	7	9	1	5
C4	1	1	5	1

Respondent 11

	C1	C2	C3	C4
C4	5	7	1	1
C2	5	1	7	7

Respondent 12

	C1	C2	C3	C4
C4	7	3	3	1
C3	1	7	1	3

Respondent 13

	C1	C2	C3	C4
C2	5	1	9	1
C4	5	1	3	1

Respondent 14

	C1	C2	C3	C4
C1	1	5	9	1
C3	9	5	1	5

Respondent 15

	C1	C2	C3	C4
C3	7	7	1	9
C1	1	3	7	9

Respondent 16

	C1	C2	C3	C4
C1	1	5	7	7
C4	7	3	1	1

Respondent 17

	C1	C2	C3	C4
C1	1	1	3	9
C2	1	1	5	1

Respondent 18

	C1	C2	C3	C4
C1	1	5	7	7
C4	7	3	1	1

Respondent 19

	C1	C2	C3	C4
C4	9	7	3	1
C3	5	5	1	3

Respondent 20

	C1	C2	C3	C4
C4	7	3	1	1
C4	7	3	1	1

Respondent 21

	C1	C2	C3	C4
C1	1	1	9	7
C3	9	9	1	3

Respondent 22

	C1	C2	C3	C4

C2	3	1	7	9
C1	1	3	1	7

Respondent 23

	C1	C2	C3	C4
C1	1	5	7	7
C4	7	3	1	1

Respondent 24

	C1	C2	C3	C4
C1	1	7	1	1
C4	1	9	9	1

Respondent 25

	C1	C2	C3	C4
C1	1	9	9	1
C4	1	3	5	1

Respondent 26

	C1	C2	C3	C4
C1	1	7	1	1
C4	1	9	9	1

Respondent 27

	C1	C2	C3	C4
C1	1	5	7	7
C4	7	3	1	1

Respondent 28

	C1	C2	C3	C4
C4	1	5	9	1
C1	1	1	1	1
