# Supplementary material for: A navigational risk evaluation of ferry transport: Continuous risk management matrix based on fuzzy Best-Worst Method
Source: PLoS One. 2024 Sep 3;19(9):e0309667. doi: 10.1371/journal.pone.0309667 (PMC11371230; doi:10.1371/journal.pone.0309667)
Supplement: Attachment — Submitted filename: 2. Responses.Long.PlosOne.pdf [file pone.0309667.s002.pdf]

Dear Editor-in-Chief and Reviewers,

Thank you for giving us the opportunity to submit a revised draft of our manuscript entitled "An assessment model of eco-efficiency for container terminals within a port" to Economics of Transportation. We appreciate the time and effort that you and the reviewers have dedicated to providing your valuable feedback on our manuscript. We are grateful to the reviewers for their insightful comments on our paper. We have been able to incorporate changes to reflect most of the suggestions provided by the reviewers. We have highlighted the changes within the manuscript. Here is a point-by-point response to the Editors' and reviewers' comments and concerns.

Reviewer #1:

This paper evaluates the navigational risk of ferry transport by a continuous risk management matrix based on the fuzzy Best-Worst Method. I think the authors have written an interesting paper dealing with an important topic. The overall representation of this paper is technically sound. I have, however, a few comments and suggestions for them:

Comment # 1. The abstract should be modified. It is therefore recommended that the abstract be carefully rewritten to effectively demonstrate the necessity, novelty, and contribution of the research, as well as highlight its major findings. Write the whole abstract in a narrative form rather than itemize.

Answer: Thanks so much for your suggestion. We already revised the abstract (Seeing **Rows 20-31**).

Comment # 2. I suggest summarizing in a table with challenges and proposed solutions by the different authors, their strengths and weaknesses. This will immediately make clear the new proposal's need and the evolution that it represents.

Answer: Thanks so much for your suggestion. One table was inserted in the literature review (Seeing **Rows 205-207** and **Rows 625-626**).

Comment # 3. The motivation for this is not highlighted in the manuscript. The manuscript's value is hidden from the reader. It needs to be emphasized in a paragraph clearly.

Answer: Thanks so much for your comment. The motivation for this paper is as follows:

First, the safety of ferry transportation has attracted much concern from governments. On top of that, recent accidents necessitate a comprehensive approach to mitigate ferry navigation-related risks. Second, ferry transport has currently witnessed numerous fatal accidents due to unsafe navigation. Thus, these accidents not only emphasize the susceptibility of ferry transportation but also stress the crucial necessity for mitigating risks and consistently enhancing safety measures in ferry navigation. Third, TRMM has been adopted extensively to assess risks in ferry transport. Nonetheless, TRMM has some drawbacks that should be overcome. Therefore, CRMM is proposed to deal with this shortcoming. Fourth, compared with the classic tools of MCDA, the advantage of BWM is fewer PCs, thus increasing its application. Additionally,

DMs' subjective assessment is arguably uncertain and imprecise. Accordingly, the theory of the fuzzy set is incorporated into BWM to allow for the representation of degrees of such uncertainty and vagueness.

According to your suggestion, we summarized paper motivations in one paragraph (Seeing **Rows 82-92**).

Comment # 4. The main contribution of the manuscript should be listed in bullets under the Introduction section.

Answer: Thanks so much for your comment. The main contributions of the manuscript were listed in bullets under the Introduction section (Seeing **Rows 99-111**).

Comment # 5. Why triangular fuzzy best worst method is utilized? Give reasons.

Answer: Thanks so much for your comment. We would like to explain as follows:

It has been argued that risk evaluation in ferry transport is characterized as a multi-criteria decision analysis (MCDA) problem. In the case of ferry transport, where various factors contribute to the complexity of risk, MCDA allows decision-makers (DMs) to consider multiple criteria simultaneously when assessing risks. Nevertheless, some of the most common tools of MCDA, such as AHP, ANP, and SAW, require numerous pairwise comparisons (PCs) of risks, thereby not only weakening their practical application, but also increasing the inconsistency of PCs. To cope with this challenge, the Best-Worst Method (BWM) developed by Rezaei (2015) has been adopted extensively to solve MCDA. Compared with the classic tools of MCDA, the primary strength of BWM is fewer PCs, thus prone to obtaining DMs' judgment and boosting the consistency of subjective evaluation. Additionally, it is illustrated that DMs' subjective assessment is often uncertain and imprecise. Accordingly, the theory of the fuzzy set is incorporated into BWM to allow for the representation of degrees of such uncertainty and vagueness.

By the way, please seeing such explanation in our manuscript from **Rows 69-81**.

Comment # 6. It is imperative to compare the proposed method with the literature. The main advantage of the proposed method should be discussed comparatively with other existing solutions.

Answer: Thanks so much for your comment. We inserted one paragraph to discuss about the main advantage of the proposed method in comparison with existing methods (Seeing **Rows 384-392**).

Comment # 7. It is noteworthy that your approach your method could be extended to various real-life decision-making problems, such as the 'bio-medical waste management, plastic waste management, renewable energy sources', and more. https://doi.org/10.1016/j.eswa.2023.120082; https://doi.org/10.1016/j.asoc.2023.110516; https://doi.org/10.1016/j.asoc.2024.111495. Include this in the future scope of the conclusion section with proper citation of relevant references.

Answer: Thanks so much for your comment. We revised our manuscript by referring some of your suggestions (Seeing **Rows 416-419**)

Reviewer #2:

Comment # 1. The paper is of importance in terms of merging continuous risk matrix and Fuzzy BWM. But I wonder how the authors generate the Figure 2. I think more details needed to explain how they structured the x-and y-axis values. What is the continuous function here?

Answer: Thanks so much for your comment. We would like to explain as follows:

a) How the authors generate the Figure 2: From Table 5 of our manuscript, we calculated risk value (RVs) of risk factors (RFs) using Equation (9). As a result, the risk value of RFs is found and exhibited in the second-to-last column of Table 6. Then, this study utilized the "ggRepel" package in Rstudio to visually represent the continuous risk management matrix (CRMM), which is precisely Figure 2.

b) More details needed to explain how they structured the x-and y-axis values: In Figure 2, the x-axis present probability (%) and the y-axis stand for severity (%) of risk factors. The more probability and severity, the more risk the risk factors are facing to. More specifically, 20 risk factors are divided into 4 groups: L (low), M (medium), H (high), and E (extreme) risk zones. Also, three risk factors located in the E zone include VD4, EE4, and PR3. In other words, ferry operators should pay more attention to such risk factors than others in the context of limited resources.

c) What is the continuous function here: To assess risks of the transport systems, the relevant research normally adopts the discrete risk management matrix (DRMM). Yet, DRMM might fail to identify true risk factors. For example, four images below present theoretical and empirical case of DRMM and CRMM:

Theory:

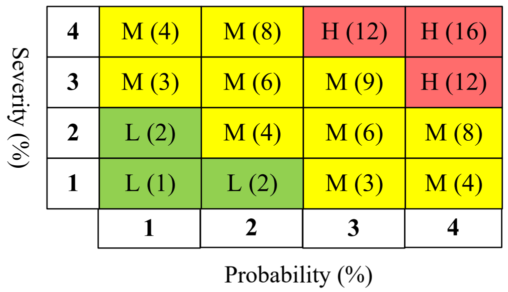

Figure a.

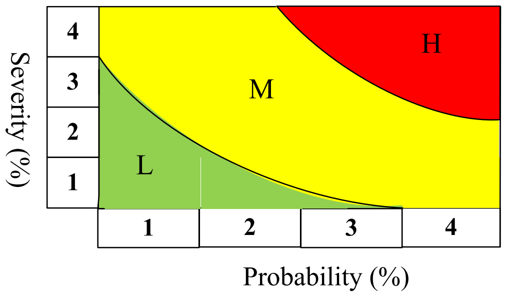

Figure b.

Empirical case:

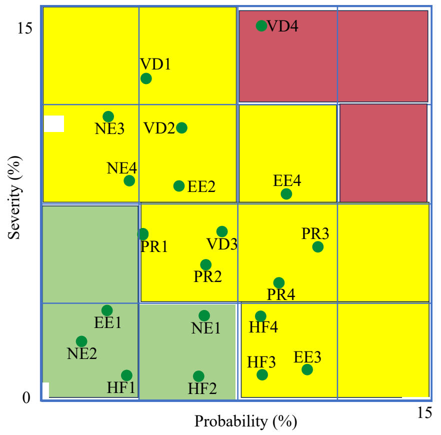

Figure c.

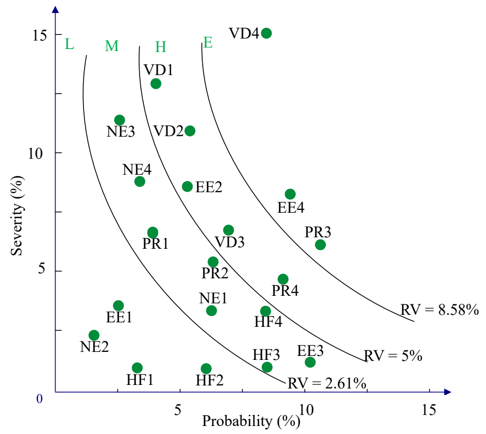

Figure d.

Theoretically, DRMM is visualized in Figure a, while CRMR is seen in Figure b. In the meantime, for the empirical case, DRMM is shown in Figure c, while CRMR is exhibited in Figure d. Evidently, the proposed method determined three extreme risk factors for prioritizing the allocation of scarce resources while DRMM only figured out one. In other words, CRMR can pinpoint risk factors better than traditional risk matrix.

We revised our manuscript by referring some of your suggestions (Seeing **Rows 323-326**).

Comment # 2. Going on from the end of comment 1, how they determine the four categories? Why four?

Answer: Thanks so much for your comment. We would like to explain as follows:

a) How authors determined the four categories: From Table 5 of our manuscript, we calculated risk value (RVs) of risk factors (RFs) using Equation (9). As a result, the risk value of RFs was found and exhibited in the second-to-last column of Table 6. Now, we had 20 risk factors with their risk values of 5%. Then, risk factors with $5\% \le RVs \le 12.43\%$ were averaged to ascertain extreme-and high-risk factors. Next, risk factors with $0.71\% \le RVs < 5\%$ were averaged to find medium-and low-risk factors.

b) Why we just categorized four: In the traditional risk matrix (i.e, DRMM), the relevant research prefers to classify risk factors into three categories, as seen Figure a, as mentioned earlier. Yet, DRMM might fail to identify true risk factors. Accordingly, our study divided risk factors into four groups to deeply understand risk factors. As a result, the proposed method (i.e., CRMR) determined three extreme risk factors for prioritizing the allocation of scarce resources while DRMM only figured out one.

Comment # 3. Why the authors prefer fuzzy version of BWM? Is there a special reason or not? Please explain in detail.

Answer: Thanks so much for your comment. We would like to explain as follows:

It has been argued that risk evaluation in ferry transport is characterized as a multi-criteria decision analysis (MCDA) problem. In the case of ferry transport, where various factors contribute to the complexity of risk, MCDA allows decision-makers (DMs) to consider multiple criteria simultaneously when assessing risks. Nevertheless, some of the most common tools of MCDA, such as AHP, ANP, and SAW, require numerous pairwise comparisons (PCs) of risks, thereby not only weakening their practical application, but also increasing the inconsistency of PCs. To cope with this challenge, the Best-Worst Method (BWM) developed by Rezaei (2015) has been adopted extensively to solve MCDA. Compared with the classic tools of MCDA, the primary strength of BWM is fewer PCs, thus prone to obtaining DMs' judgment and boosting the consistency of subjective evaluation. Additionally, it is illustrated that DMs' subjective assessment is often uncertain and imprecise. Accordingly, the theory of the fuzzy set is incorporated into BWM to allow for the representation of degrees of such uncertainty and vagueness.

By the way, please seeing **Rows 69-81** for more information.

Comment # 4. I think a flow chart is required to easily understand the integration between CRMM and fuzzy BWM. More theoretical information is needed for CRMM.

Answer: Thanks so much for your suggestion. We already inserted one flowchart to explain the integration between CRMM and fuzzy BWM (Seeing **Rows 209-216**). Besides, more theoretical information for CRMM was added (Seeing **Rows 275-279**).

Comment # 5. Authors provided a consistency computation in their method part but there is no output for this in the application. Please provide your results regarding consistency.

Answer: Thanks so much for your suggestion. We would like to explain that:

For the application of fuzzy BWM, we assessed consistency of every judgment for every respondent. It is worth noting to emphasise that the nature of BWM is that we cannot average expert's rating. Please seeing Rezaei (2015) [1] and Mi et al. (2019)[2] for more information. As a result, we were only able to calculate the consistency ratio (CR) for each expert in terms of each dimension (criterion).

With reference to your request, the table below shows the consistency ratio of ratings in the FO-VN case. Note that all $CR < 10\%$ illustrate the consistency of judgments.

Respondent	Dimension/Criterion	Probability			Severity		
		$\tilde{\varepsilon}$	CI	CR	$\tilde{\varepsilon}$	CI	CR
Respondent # 1	Layer 1: Dimension	0.6173	6.69	9.23%	0.2318	3.8	6.10%
	Layer 2:	0.1557	3.8	4.10%	0.1079	5.29	2.04%
	HF	0.2646	5.29	5.00%	0.1925	3.8	5.07%
	NE	0.0545	5.29	1.03%	0.2117	3	7.06%
	PR	0.2288	3.8	6.02%	0.1605	5.29	3.03%
	VD	0.3181	3.8	8.37%	0.0674	6.69	1.01%
	EE	0.2305	3.8	6.07%	0.1347	6.69	2.01%
Respondent # 2	Layer 1: Dimension	0.0311	3	1.04%	0.0620	3	2.07%
	Layer 2:	0.3212	5.29	6.07%	0.0734	6.69	1.10%
	HF	0.4699	6.69	7.02%	0.0859	8.08	1.06%
	NE	0.2687	3.8	7.07%	0.6517	8.08	8.07%
	PR	0.5131	5.29	9.70%	0.0542	5.29	1.02%
	VD	0.2419	3	8.06%	0.6503	8.08	8.05%
	EE	0.1938	3.8	5.10%	0.3428	3.8	9.02%
Respondent # 3	Layer 1: Dimension	0.5212	5.29	9.85%	0.1633	8.08	2.02%
	Layer 2:	0.4891	8.08	6.05%	0.5031	5.29	9.51%
	HF	0.8010	8.08	9.91%	0.3210	5.29	6.07%
	NE	0.3701	3.8	9.74%	0.2874	3	9.58%
	PR	0.1692	8.08	2.09%	0.6076	6.69	9.08%
	VD	0.1213	3	4.04%	0.1662	8.08	2.06%

[1] Rezaei, J. (2015). Best-worst multi-criteria decision-making method. *Omega*, *53*, 49-57.
[2] Mi, X., Tang, M., Liao, H., Shen, W., & Lev, B. (2019). The state-of-the-art survey on integrations and applications of the best worst method in decision making: Why, what, what for and what's next?. *Omega*, *87*, 205-225.

Respondent	Dimension/Criterion	Probability			Severity		
		$\tilde{\varepsilon}$	CI	CR	$\tilde{\varepsilon}$	CI	CR
	EE	0.2111	3	7.04%	0.2165	5.29	4.09%
Respondent # 4	Layer 1: Dimension	0.2662	3.8	7.00%	0.1545	3.8	4.06%
	Layer 2:	0.0904	3	3.01%	0.4871	8.08	6.03%
	HF	0.4712	6.69	7.04%	0.8010	8.08	9.91%
	NE	0.3287	8.08	4.07%	0.0326	3	1.09%
	PR	0.0783	3.8	2.06%	0.2120	3	7.07%
	VD	0.0772	3.8	2.03%	0.2689	5.29	5.08%
	EE	0.3215	5.29	6.08%	0.1145	3.8	3.01%
Respondent # 5	Layer 1: Dimension	0.8012	8.08	9.92%	0.2125	3	7.08%
	Layer 2:	0.1358	6.69	2.03%	0.3710	3.8	9.76%
	HF	0.5718	8.08	7.08%	0.7299	8.08	9.03%
	NE	0.1632	5.29	3.08%	0.6670	6.69	9.97%
	PR	0.5130	5.29	9.70%	0.2681	3.8	7.05%
	VD	0.5714	8.08	7.07%	0.2036	6.69	3.04%
	EE	0.2728	3	9.09%	0.2431	8.08	3.01%
Respondent # 6	Layer 1: Dimension	0.1062	5.29	2.01%	0.3402	6.69	5.09%
	Layer 2:	0.0911	3	3.04%	0.3728	3.8	9.81%
	HF	0.4025	6.69	6.02%	0.1810	3	6.03%
	NE	0.1542	3.8	4.06%	0.4054	6.69	6.06%
	PR	0.1206	3	4.02%	0.6510	6.69	9.73%
	VD	0.2467	8.08	3.05%	0.4060	8.08	5.03%
	EE	0.6052	6.69	9.05%	0.1589	5.29	3.00%
Respondent # 7	Layer 1: Dimension	0.1108	5.29	2.09%	0.1817	3	6.06%
	Layer 2:	0.3451	3.8	9.08%	0.3433	3.8	9.03%
	HF	0.2717	3	9.06%	0.2436	8.08	3.01%
	NE	0.2657	5.29	5.02%	0.3409	6.69	5.10%
	PR	0.4909	8.08	6.08%	0.4043	6.69	6.04%
	VD	0.4255	5.29	8.04%	0.2256	3	7.52%
	EE	0.0852	8.08	1.05%	0.2317	3.8	6.10%
Respondent # 8	Layer 1: Dimension	0.0614	3	2.05%	0.7289	8.08	9.02%
	Layer 2:	0.3385	6.69	5.06%	0.2711	3	9.04%
	HF	0.0410	3.8	1.08%	0.1919	3.8	5.05%
	NE	0.0908	3	3.03%	0.5098	5.29	9.64%
	PR	0.3436	3.8	9.04%	0.0871	8.08	1.08%
	VD	0.0327	3	1.09%	0.2498	8.08	3.09%
	EE	0.6171	6.69	9.22%	0.1933	3.8	5.09%
Respondent # 9	Layer 1: Dimension	0.5394	6.69	8.06%	0.4071	6.69	6.08%
	Layer 2:	0.0850	8.08	1.05%	0.1642	8.08	2.03%
	HF	0.6272	6.69	9.38%	0.5099	5.29	9.64%
	NE	0.1547	3.8	4.07%	0.5672	8.08	7.02%
	PR	0.0902	3	3.01%	0.5366	6.69	8.02%

Respondent	Dimension/Criterion	Probability			Severity		
		$\tilde{\varepsilon}$	CI	CR	$\tilde{\varepsilon}$	CI	CR
	VD	0.6045	6.69	9.04%	0.2202	3	7.34%
	EE	0.0701	6.69	1.05%	0.6115	6.69	9.14%
Respondent # 10	Layer 1: Dimension	0.1554	3.8	4.09%	0.3245	8.08	4.02%
	Layer 2:	0.2314	3.8	6.09%	0.2054	6.69	3.07%
	HF	0.3371	6.69	5.04%	0.1098	5.29	2.08%
	NE	0.1204	3	4.01%	0.2715	3	9.05%
	PR	0.2146	5.29	4.06%	0.7273	8.08	9.00%
	VD	0.1062	5.29	2.01%	0.1508	3	5.03%
	EE	0.7319	8.08	9.06%	0.1073	5.29	2.03%
Respondent # 11	Layer 1: Dimension	0.7292	8.08	9.02%	0.4044	6.69	6.04%
	Layer 2:	0.4279	5.29	8.09%	0.0795	3.8	2.09%
	HF	0.2011	6.69	3.01%	0.0555	5.29	1.05%
	NE	0.3207	5.29	6.06%	0.4033	6.69	6.03%
	PR	0.1504	3	5.01%	0.4299	5.29	8.13%
	VD	0.2416	3	8.05%	0.4042	6.69	6.04%
	EE	0.1804	3	6.01%	0.1143	3.8	3.01%
Respondent # 12	Layer 1: Dimension	0.1818	3	6.06%	0.3393	6.69	5.07%
	Layer 2:	0.0768	3.8	2.02%	0.3184	5.29	6.02%
	HF	0.2701	6.69	4.04%	0.2064	6.69	3.09%
	NE	0.3067	3.8	8.07%	0.2568	3	8.56%
	PR	0.1829	3	6.10%	0.0320	3	1.07%
	VD	0.3070	3.8	8.08%	0.2703	3	9.01%
	EE	0.2494	8.08	3.09%	0.2703	3	9.01%
Respondent # 13	Layer 1: Dimension	0.4739	6.69	7.08%	0.1152	3.8	3.03%
	Layer 2:	0.1927	3.8	5.07%	0.3202	5.29	6.05%
	HF	0.4040	6.69	6.04%	0.2721	3	9.07%
	NE	0.2703	3	9.01%	0.6789	8.08	8.40%
	PR	0.6607	6.69	9.88%	0.1523	3.8	4.01%
	VD	0.1208	3	4.03%	0.2130	3	7.10%
	EE	0.0868	8.08	1.07%	0.0390	3.8	1.03%
Respondent # 14	Layer 1: Dimension	0.2416	3	8.05%	0.2736	6.69	4.09%
	Layer 2:	0.2680	3.8	7.05%	0.5131	5.29	9.70%
	HF	0.1354	6.69	2.02%	0.7414	8.08	9.18%
	NE	0.4883	8.08	6.04%	0.1101	5.29	2.08%
	PR	0.8014	8.08	9.92%	0.2486	8.08	3.08%
	VD	0.3381	6.69	5.05%	0.5712	8.08	7.07%
	EE	0.0543	5.29	1.03%	0.0320	3	1.07%
Respondent # 15	Layer 1: Dimension	0.3730	5.29	7.05%	0.3385	6.69	5.06%
	Layer 2:	0.4241	5.29	8.02%	0.2338	3	7.79%
	HF	0.2151	5.29	4.07%	0.3381	3.8	8.90%
	NE	0.3457	3.8	9.10%	0.1629	5.29	3.08%

Respondent	Dimension/Criterion	Probability			Severity		
		$\tilde{\varepsilon}$	CI	CR	$\tilde{\varepsilon}$	CI	CR
	PR	0.2426	3	8.09%	0.1548	3.8	4.07%
	VD	0.0606	3	2.02%	0.2683	6.69	4.01%
	EE	0.1218	3	4.06%	0.0571	5.29	1.08%
Respondent # 16	Layer 1: Dimension	0.2681	6.69	4.01%	0.2064	6.69	3.09%
	Layer 2:	0.1512	3	5.04%	0.3401	6.69	5.08%
	HF	0.5695	8.08	7.05%	0.4014	6.69	6.00%
	NE	0.3683	3.8	9.69%	0.3483	3.8	9.17%
	PR	0.2117	5.29	4.00%	0.1540	3.8	4.05%
	VD	0.0611	3	2.04%	0.4736	6.69	7.08%
	EE	0.2483	8.08	3.07%	0.1627	8.08	2.01%
Respondent # 17	Layer 1: Dimension	0.4919	8.08	6.09%	0.2457	8.08	3.04%
	Layer 2:	0.7121	8.08	8.81%	0.3713	5.29	7.02%
	HF	0.2717	3	9.06%	0.1627	8.08	2.01%
	NE	0.4880	8.08	6.04%	0.3456	3.8	9.09%
	PR	0.3732	5.29	7.05%	0.3429	3.8	9.02%
	VD	0.1637	5.29	3.09%	0.3293	8.08	4.08%
	EE	0.7152	8.08	8.85%	0.2285	3.8	6.01%
Respondent # 18	Layer 1: Dimension	0.3719	5.29	7.03%	0.2130	3	7.10%
	Layer 2:	0.4927	8.08	6.10%	0.0604	3	2.01%
	HF	0.3451	3.8	9.08%	0.2035	6.69	3.04%
	NE	0.1635	8.08	2.02%	0.2449	8.08	3.03%
	PR	0.1509	3	5.03%	0.5660	8.08	7.00%
	VD	0.1148	3.8	3.02%	0.2685	5.29	5.07%
	EE	0.0540	5.29	1.02%	0.4730	6.69	7.07%
Respondent # 19	Layer 1: Dimension	0.1507	3	5.02%	0.6370	6.69	9.52%
	Layer 2:	0.1505	3	5.02%	0.2729	3	9.10%
	HF	0.1341	6.69	2.00%	0.6500	8.08	8.04%
	NE	0.4236	5.29	8.01%	0.1819	3	6.06%
	PR	0.6672	6.69	9.97%	0.0626	3	2.09%
	VD	0.1399	6.69	2.09%	0.2292	3.8	6.03%
	EE	0.1685	8.08	2.09%	0.3440	3.8	9.05%
Respondent # 20	Layer 1: Dimension	0.1602	5.29	3.03%	0.4717	6.69	7.05%
	Layer 2:	0.4068	6.69	6.08%	0.1616	8.08	2.00%
	HF	0.2287	3.8	6.02%	0.0926	3	3.09%
	NE	0.0774	3.8	2.04%	0.2706	3	9.02%
	PR	0.4715	6.69	7.05%	0.4737	6.69	7.08%
	VD	0.1634	8.08	2.02%	0.2112	3	7.04%
	EE	0.1070	5.29	2.02%	0.3385	6.69	5.06%
Respondent # 21	Layer 1: Dimension	0.3303	8.08	4.09%	0.0604	3	2.01%
	Layer 2:	0.0571	5.29	1.08%	0.3205	5.29	6.06%
	HF	0.5360	6.69	8.01%	0.1356	6.69	2.03%

Respondent	Dimension/Criterion	Probability			Severity		
		$\tilde{\varepsilon}$	CI	CR	$\tilde{\varepsilon}$	CI	CR
	NE	0.4256	5.29	8.05%	0.0413	3.8	1.09%
	PR	0.3259	8.08	4.03%	0.3725	5.29	7.04%
	VD	0.6066	6.69	9.07%	0.2897	3	9.66%
	EE	0.0704	6.69	1.05%	0.2103	3	7.01%
Respondent # 22	Layer 1: Dimension	0.3266	8.08	4.04%	0.1229	3	4.10%
	Layer 2:	0.6482	8.08	8.02%	0.1105	5.29	2.09%
	HF	0.3783	3.8	9.96%	0.2410	3	8.03%
	NE	0.7291	8.08	9.02%	0.1076	5.29	2.03%
	PR	0.1158	3.8	3.05%	0.0618	3	2.06%
	VD	0.4792	5.29	9.06%	0.1093	5.29	2.07%
	EE	0.5355	6.69	8.00%	0.2661	5.29	5.03%
Respondent # 23	Layer 1: Dimension	0.6374	6.69	9.53%	0.0416	3.8	1.09%
	Layer 2:	0.3213	5.29	6.07%	0.1806	3	6.02%
	HF	0.2658	5.29	5.02%	0.4079	6.69	6.10%
	NE	0.5231	5.29	9.89%	0.2430	3	8.10%
	PR	0.3455	3.8	9.09%	0.2771	3	9.24%
	VD	0.2434	8.08	3.01%	0.4867	8.08	6.02%
	EE	0.2901	3	9.67%	0.4812	5.29	9.10%
Respondent # 24	Layer 1: Dimension	0.1083	5.29	2.05%	0.6538	8.08	8.09%
	Layer 2:	0.0416	3.8	1.09%	0.1521	3	5.07%
	HF	0.2009	6.69	3.00%	0.2105	3	7.02%
	NE	0.7296	8.08	9.03%	0.2604	3	8.68%
	PR	0.0301	3	1.00%	0.3182	3.8	8.37%
	VD	0.2474	8.08	3.06%	0.1210	3	4.03%
	EE	0.6592	6.69	9.85%	0.0733	6.69	1.10%
Respondent # 25	Layer 1: Dimension	0.4037	6.69	6.03%	0.0605	3	2.02%
	Layer 2:	0.4729	6.69	7.07%	0.2317	3.8	6.10%
	HF	0.1615	5.29	3.05%	0.1591	5.29	3.01%
	NE	0.7713	8.08	9.55%	0.7610	8.08	9.42%
	PR	0.2159	5.29	4.08%	0.2157	5.29	4.08%
	VD	0.7811	8.08	9.67%	0.4717	6.69	7.05%
	EE	0.4704	6.69	7.03%	0.4717	6.69	7.05%
Respondent # 26	Layer 1: Dimension	0.4054	6.69	6.06%	0.3712	5.29	7.02%
	Layer 2:	0.4112	8.08	5.09%	0.1067	5.29	2.02%
	HF	0.3223	5.29	6.09%	0.5132	5.29	9.70%
	NE	0.2694	6.69	4.03%	0.0553	5.29	1.04%
	PR	0.0918	3	3.06%	0.2425	3	8.08%
	VD	0.0603	3	2.01%	0.5686	8.08	7.04%
	EE	0.1601	5.29	3.03%	0.1390	6.69	2.08%
Respondent # 27	Layer 1: Dimension	0.5372	6.69	8.03%	0.1371	6.69	2.05%
	Layer 2:	0.3433	3.8	9.03%	0.5657	8.08	7.00%

**Respondent**	**Dimension/Criterion**	**Probability**			**Severity**		
		$\tilde{\varepsilon}$	**CI**	**CR**	$\tilde{\varepsilon}$	**CI**	**CR**
	HF	0.6520	8.08	8.07%	0.1380	6.69	2.06%
	NE	0.2405	3	8.02%	0.3049	3.8	8.02%
	PR	0.7329	8.08	9.07%	0.3725	5.29	7.04%
	VD	0.0901	3	3.00%	0.5716	8.08	7.07%
	EE	0.1230	3	4.10%	0.0619	3	2.06%
Respondent # 28	Layer 1: Dimension	0.3189	5.29	6.03%	0.1141	3.8	3.00%
	Layer 2:	0.2283	3.8	6.01%	0.0760	3.8	2.00%
	HF	0.0919	3	3.06%	0.2730	6.69	4.08%
	NE	0.1342	6.69	2.01%	0.1061	5.29	2.01%
	PR	0.4056	8.08	5.02%	0.2111	3	7.04%
	VD	0.3075	3.8	8.09%	0.7809	8.08	9.67%
	EE	0.2123	5.29	4.01%	0.1169	3.8	3.08%

Academic Editor:

Comment # 1. Please ensure that your manuscript meets PLOS ONE's style requirements, including those for file naming.

Answer: Thanks so much for your comments. We check across our manuscript to ensure it meets PLOS ONE's style requirements.

Comment # 2. Please include your full ethics statement in the 'Methods' section of your manuscript file. In your statement, please include the full name of the IRB or ethics committee who approved or waived your study, as well as whether or not you obtained informed written or verbal consent. If consent was waived for your study, please include this information in your statement as well.

Answer: Thanks so much for your comments. Consent was waived for our study. So, we included this information in our statement, as the attached file.

Comment # 3. We note that Figure 1 in your submission contain [map/satellite] images which may be copyrighted.

Answer: Thanks so much for your comments. We deleted this figure out of our paper.

One more time, the authors are very grateful to the reviewers to check the paper so carefully and propose so many constructive suggestions to improve the paper.

Best regards.

The 1st author name: Linh Thi Pham

The 2nd author name: Long Van Hoang
